# Enhanced Immunosuppression of T Cells by Sustained Presentation of Bioactive Interferon‐γ Within Three‐Dimensional Mesenchymal Stem Cell Constructs

**DOI:** 10.5966/sctm.2016-0044

**Published:** 2016-08-08

**Authors:** Joshua A. Zimmermann, Marian H. Hettiaratchi, Todd C. McDevitt

**Affiliations:** ^1^ The Wallace H. Coulter Department of Biomedical Engineering, Georgia Institute of Technology & Emory University, Atlanta, Georgia, USA; ^2^ Gladstone Institute of Cardiovascular Disease, San Francisco, California, USA; ^3^ Department of Bioengineering and Therapeutic Sciences, University of California–San Francisco, San Francisco, California, USA

**Keywords:** Mesenchymal stromal cells, Immunomodulation, Spheroids, Cellular, Indoleamine 2,3‐dioxygenase

## Abstract

The immunomodulatory activity of mesenchymal stem/stromal cells (MSCs) to suppress innate and adaptive immune responses offers a potent cell therapy for modulating inflammation and promoting tissue regeneration. However, the inflammatory cytokine milieu plays a critical role in stimulating MSC immunomodulatory activity. In particular, interferon‐γ (IFN‐γ)‐induced expression of indoleamine 2,3‐dioxygenase (IDO) is primarily responsible for MSC suppression of T‐cell proliferation and activation. Although pretreatment with IFN‐γ is commonly used to prime MSCs for immunomodulatory activity prior to transplantation, the transient effects of pretreatment may limit the potential of MSCs to potently modulate immune responses. Therefore, the objective of this study was to investigate whether microparticle‐mediated presentation of bioactive IFN‐γ within three‐dimensional spheroidal MSC aggregates could precisely regulate and induce sustained immunomodulatory activity. Delivery of IFN‐γ via heparin‐microparticles within MSC aggregates induced sustained IDO expression during 1 week of culture, whereas IDO expression by IFN‐γ‐pretreated MSC spheroids rapidly decreased during 2 days. Furthermore, sustained IDO expression induced by IFN‐γ‐loaded microparticles resulted in an increased and sustained suppression of T‐cell activation and proliferation in MSC cocultures with CD3/CD28‐activated peripheral blood mononuclear cells. The increased suppression of T cells by MSC spheroids containing IFN‐γ‐loaded microparticles was dependent on induction of IDO and supported by affecting monocyte secretion from pro‐ to anti‐inflammatory cytokines. Altogether, microparticle delivery of IFN‐γ within MSC spheroids provides a potent means of enhancing and sustaining immunomodulatory activity to control MSC immunomodulation after transplantation and thereby improve the efficacy of MSC‐based therapies aimed at treating inflammatory and immune diseases. Stem Cells Translational Medicine *2017;6:223–237*

**Significance Statement**

This study demonstrates that biomaterial‐based presentation of cytokines within spheroidal mesenchymal stem/stromal cell (MSC) aggregates provides a means of locally concentrating and sustaining presentation of cytokines to potentiate MSC immunomodulatory activity. Overall, this approach could aid in overcoming the limitations of transient pretreatment strategies by continuously presenting cytokines within the MSC microenvironment and thereby maximizing the immunomodulatory potential of transplanted MSCs. Furthermore, this approach provides a means of inducing potent MSC immunomodulation, irrespective of unknown or ill‐defined environmental inflammatory milieu, such as in chronic inflammation or when administered with immunosuppressants. Altogether, biomaterial‐based engineering of the MSC microenvironment provides a means of controlling the function of transplanted cells to specifically direct their therapeutic activity and may improve MSC‐based therapies aimed at treating a number of inflammatory and immune diseases.

## Introduction

Mesenchymal stem/stromal cells (MSCs) are potent modulators of inflammatory and immune responses because of their ability to secrete soluble paracrine factors that regulate both innate and adaptive immunity and repolarize cells from proinflammatory to anti‐inflammatory or proresolving phenotypes. Initial work first demonstrated that MSCs potently inhibit T‐cell proliferation and activation upon T‐cell receptor stimulation in vitro 1, 2. These initial observations motivated the use of MSCs as a potential therapeutic agent in inflammatory and immune diseases dominated by a pathogenic T‐cell response and spurred research to better understand the interactions of MSCs and the immune system. Since then, MSCs have been demonstrated to regulate numerous leukocyte populations, including T cells, B cells 3, macrophages 4, dendritic cells 5, and natural killer cells 6. Moreover, this ability of MSCs to modulate multiple components that contribute to the complexity of an immune response further motivated the use of MSCs to treat diseases such as graft‐versus‐host disease, inflammatory bowel disease, and autoimmune disorders 7. However, the ability of MSCs to suppress T‐cell activation and alter T‐cell polarization has remained the primary focus of much of MSC immunomodulatory research, and the soluble signals and pathways governing the interaction between MSCs and T cells are well characterized in comparison with other leukocyte populations.

Multiple paracrine and immunomodulatory factors play a role in MSC‐mediated suppression of T‐cell proliferation and activation, including indoleamine 2,3‐dioxygenase (IDO), prostaglandin E2 (PGE_2_), transforming growth factor β 1 (TGF‐β1), human leukocyte antigen‐G5, heme oxygenase 1, CC chemokine ligand 2, Fas ligand (FasL), and programmed death ligand 1 and 2 (PDL1 and PDL2) 8 9 10 11 12 13 14 15 16. Furthermore, the inhibition of individual MSC‐expressed factors significantly reduces the potency of MSC immunomodulation in vitro and in vivo, suggesting that the coordinated action of the immunomodulatory secretome of MSCs is necessary to regulate complex immune responses. However, the expression of IDO is critically necessary for MSC suppression of T‐cell proliferation, and the variable amounts of IDO expression between MSC donors are strongly correlated with the ability to suppress T‐cell responses 17, 18. IDO inhibits proliferation of T cells through tryptophan depletion 19, and the production of kynurenine, a tryptophan metabolite, that suppresses T‐cell proliferation through activation of the stress response kinase GCN2 20, 21. Additionally, IDO expression induces naïve CD4^+^CD25^−^ T‐cell maturation into CD4^+^CD25^+^FOXP3^+^ regulatory T cells 22 and aids in the induction of immune tolerance 23. Although IDO is not constitutively expressed by resting MSCs, its expression is strongly induced by exposure of MSCs to interferon‐γ (IFN‐γ). IFN‐γ licensing alone or in combination with other cytokines (tumor necrosis factor [TNF]‐α, interleukin [IL]‐1β) is crucial for MSC immunomodulatory activity. Thus, MSC immunomodulation is highly dependent on the local inflammatory milieu to activate IDO and cytokine expression.

Because of the dependency of MSC immunomodulation on the local cytokine milieu, the efficacy of MSC‐based cellular therapies is highly dependent on the in vivo environment that they are exposed to after injection. The environment may be highly variable on the basis of the individual and disease being treated, the stage of inflammation, and the site of MSC transplantation. For example, high concentrations of inflammatory cytokines that often accompany acute inflammatory responses stimulate MSC immunomodulation 24. However, the low levels of cytokines observed in chronic inflammation may not be sufficient to induce MSC immunomodulatory activity, thereby limiting the ability of MSCs to regulate inflammation of certain disease states. Additionally, MSCs are typically infused intravenously, and the majority of cells are entrapped within the capillary beds of the lung tissue, distant from sites of inflammation 25. Entrapped MSCs are then rapidly cleared from the body within several days, limiting their residency time and effective window for immunomodulatory activity. Interestingly, the physical entrapment of MSCs in the lung causes the formation of aggregates of MSCs within the lung tissue, which results in upregulation of immunomodulatory factors such as PGE_2_ and TNF‐stimulated gene 6, even in the absence of any cytokine priming 26 27 28. Although i.v.‐infused MSCs are capable of modulating immune responses in numerous disease models and human patients, presumably through systemic effects of MSCs entrapped within the lung, the limited retention time and lack of exposure to local inflammation may limit the overall immunomodulatory efficacy of transplanted MSCs.

To overcome these limitations, the use of local transplantation of MSCs as preformed aggregates may improve retention of the cells at the site of interest, expose MSCs to the local inflammatory environment, and promote increased expression of immunomodulatory factors. Delivery of MSCs as aggregates is thought to preserve cell‐cell and cell‐matrix contacts within the aggregate structures, thereby preventing cell loss due to anoikis and resulting in better engraftment in host tissue 29, 30. Although systemic infusion of aggregates would not be possible because of the likelihood of occluding blood vessels, local transplantation through a larger bore needle is feasible and may be beneficial in concentrating MSC factors at sites of inflammation as well as exposing MSCs to the local inflammatory cytokine milieu. As a potentially complementary strategy to enhance immunomodulation, MSCs are commonly pretreated with inflammatory cytokines, such as IFN‐γ, prior to intravenous infusion to augment their immunomodulatory activity during their minimal residence time after transplantation. IFN‐γ−pretreated MSCs have exhibited improved resolution of inflammation in models of colitis 31 and graft‐versus‐host disease 32, in comparison with untreated MSCs. Furthermore, pretreatment of MSC aggregates with IFN‐γ and TNF‐α further increases the immunomodulatory activity of MSC spheroids 33. However, despite the enhancement of MSC immunomodulation, the transient effects of pretreatment of MSCs may limit the potential of MSC spheroids to modulate immune responses for more than a few days in environments that do not expose the cells to high concentrations of IFN‐γ, such as in chronic inflammatory states.

To address the transient effects of pretreatment, we hypothesized that biomaterial‐based presentation of cytokines within spheroidal MSC aggregates may provide a means of locally concentrating and sustaining presentation of bioactive IFN‐γ to potentiate MSC immunomodulatory activity. Previous work from our group has demonstrated the use of microparticles (MPs) to deliver growth factors and small molecules throughout stem cell aggregates 34 35 36 37. Furthermore, microparticles consisting of cross‐linked heparin have previously been fabricated and are capable of binding a large amount of heparin binding growth factors that remain bioactive when immobilized to the particles 38. Because heparin has a high affinity for IFN‐γ (K_D_= 1–5 nM) 39, 40, can prevent proteolytic degradation of IFN‐γ 41, and can enhance IFN‐γ signaling 42, heparin microparticles were used to deliver IFN‐γ within MSC spheroids in these studies. The ability of microparticle‐delivered IFN‐γ to induce sustained immunomodulatory activity was evaluated by measuring IDO expression of MSCs in response to microparticles and pretreatment and by assessing suppression of T‐cell activation and proliferation in peripheral blood mononuclear cell (PBMC) cocultures. Altogether, this strategy offers a translatable means of controlling MSC paracrine activity posttransplantation and therefore improve the efficacy of MSC‐based treatment strategies for inflammatory and immune diseases.

## Methods

### Heparin Microparticle Synthesis

Heparin methacrylamide microparticles were fabricated, as has been previously described 38. Briefly, heparin methacrylamide was synthesized from heparin ammonium salt derived from porcine intestinal mucosa (17–19 kDa; Sigma‐Aldrich, St. Louis, MO, https://www.sigmaaldrich.com) and N‐(3‐aminopropyl) methacrylamide (APMAm; Polysciences, Warrington, PA, http://www.polysciences.com/) using 1‐ethyl‐3‐(3‐dimethylaminopropyl) carbodiimide (EDC; Thermo Scientific, Rockford, IL, https://www.thermofisher.com) and N‐hydroxysulfosuccinimide (Sulfo‐NHS; Thermo Scientific) 43. Heparin ammonium salt (325 mg) was dissolved in distilled water (200 ml) with a 10‐fold molar excess of EDC, sulfo‐NHS, and APMAm in relation to the heparin carboxyl groups. The reaction was allowed to proceed for 5 hours at room temperature and pH 6.5, before being dialyzed against 2 liters of water using 3.5 kDa molecular weight cutoff dialysis tubing (Spectrum Laboratories, Rancho Dominguez, CA, http://www.spectrumlabs.com) for 48 hours to remove excess reactants; dialysis water was exchanged during 12 hours. The remaining heparin solution was then lyophilized for 4 days and stored at −20°C.

To fabricate heparin microparticles, a water‐in‐oil emulsion technique was used to generate small droplets of heparin solution that were subsequently stabilized by thermal cross‐linking of the methacrylamide groups on the heparin species. We dissolved 55.6 mg of lyophilized heparin methacrylamide in 400 µl of phosphate‐buffered saline (PBS; Corning Mediatech, Manassas, VA, https://www.corning.com), supplemented with 18 mM of the free radical initiators ammonium persulfate (Sigma Aldrich) and tetramethylethylenediamine (Sigma Aldrich). The heparin solution was added dropwise to 60 ml of corn oil and 1 ml of polysorbate‐20 (Promega, Madison, WI, http://www.promega.com/). To form an emulsion, we homogenized the entire solution on ice at 3,000 rpm for 5 minutes by using a Polytron PT31000 homogenizer (Kinematica, Luzerne, Switzerland, http://www.kinematica.ch/). After homogenization, free radical polymerization of the methacrylamide groups was initiated by submerging the emulsion in a hot water bath (55°C) with gentle agitation under nitrogen purging for 30 minutes. The solution was then centrifuged at 3,000 rpm for 10 minutes at 4°C to obtain a pellet of microparticles. Microparticles were washed once in acetone, followed by three water washes to remove residual corn oil and loosely cross‐linked particles. The remaining microparticles were disinfected in a 70% ethanol solution for 30 minutes, followed by additional washes with sterile water, lyophilized for 2 days, and stored at 4°C prior to use.

### Heparin Microparticle Characterization

To determine the extent of IFN‐γ binding to heparin methacrylamide microparticles, we incubated approximately 1 × 10^6^ particles at room temperature in 50 µl of PBS with 0.1% bovine serum albumin and a range of IFN‐γ (10–1,000 ng; R&D Systems, Minneapolis, MN, https://www.rndsystems.com) that is typically used to stimulate MSC IDO expression 44. After 18 hours, the microparticles were centrifuged at 200× *g* for 5 minutes and the supernatant collected to determine the amount of free IFN‐γ remaining in the solution. The amount of unbound IFN‐γ was quantified by using a human IFN‐γ enzyme‐linked immunosorbent assay (ELISA kit; R&D) and compared with an equivalent amount of IFN‐γ incubated for 18 hours without microparticles to generate a loading curve for IFN‐γ binding to heparin microparticles. After the supernatant was collected to determine the amount of bound IFN‐γ, microparticles were incubated in 1 ml of Roswell Park Memorial Institute (RPMI)‐1640 media with 10% fetal bovine serum (FBS) and incubated at 37°C for 7 days in a humidified 5% CO_2_ incubator. We sampled 100 µl of the medium and replaced it with an equivalent volume each day to determine the amount of IFN‐γ released from the particles over time.

### MSC Expansion and Culture

Human bone marrow‐derived MSCs were obtained from RoosterBio Inc. (Frederick, MD, http://www.roosterbio.com/). RoosterBio MSCs demonstrated the ability to undergo adipogenic and osteogenic differentiation and expressed the accepted panel of surface markers (CD45^−^, CD34^−^, CD73^+^, CD90^+^, CD105^+^) by the manufacturer prior to use. Adipogenic and osteogenic differentiation potential were evaluated by Oil Red O and Alizarin Red staining, respectively, after 3 weeks of culture in the respective Thermo Fisher Scientific (Carlsbad, CA, https://www.thermofisher.com) differentiation kits. Additionally, MSCs were 0% CD45^+^, 0.1% CD34^+^, 98.9% CD73^+^, 99.5% CD90^+^, and 95.9% CD105^+^, as were evaluated by flow cytometry. MSCs were expanded according to the manufacturer's protocols. Briefly, 1 × 10^7^ cryopreserved MSCs were plated in 12 T225 flasks with 42 ml each of RoosterBio High‐Performance Media and incubated at 37°C for 7 days in a humidified 5% CO_2_ incubator. Media were exchanged after 4 days of culture. Cultures were passaged at 80% confluence by washing with 10 ml PBS, followed by incubation with 10 ml of TrypLE at 37°C. An equal volume of RoosterBio High‐Performance Media was added to quench TrypLE activity. Dissociated cells were then collected and centrifuged at 200× *g*. Cells were frozen in CryoStor CS5 cell cryopreservation media (STEMCELL Technologies, Vancouver, British Columbia, Canada, https://www.stemcell.com/) prior to expansion for experiments. MSCs were expanded for one or two passages from frozen stocks by plating 0.5 × 10^6^ cells in RoosterBio High‐Performance Media in 15 cm tissue culture‐treated dishes (Corning Mediatech). Media were exchanged every 3 days, and cells were passaged at 80% confluence. For all subsequent experiments, cell culture was conducted in RPMI‐1640 medium (Corning Mediatech), supplemented with 10% FBS (HyClone Laboratories, Logan, UT, https://promo.gelifesciences.com/gl/hyclone/), 1% L‐glutamine (Corning Mediatech), and 1% penicillin/streptomycin (Corning Mediatech).

### IFN‐γ‐Loaded Microparticle Bioactivity

To evaluate the bioactivity of IFN‐γ bound to heparin microparticles, we cultured adherent monolayer MSCs with heparin microparticles loaded with 0 or 333 ng of IFN‐γ per 1 × 10^6^ particles. Particles were loaded with each IFN‐γ concentration 1, 2, 4, or 7 days prior to addition of particles to adherent MSC cultures to assess the activity of microparticle‐bound IFN‐γ over time. After 18 hours of loading, particles were washed to remove excess IFN‐γ and incubated in RPMI‐1640 medium supplemented with 10% FBS at 37°C until addition of particles to adherent MSC cultures. MSCs were plated in 24‐well plates at a density of 50,000 cells/cm^2^, and after 18 hours, ∼200,000 particles were added to MSCs from each experimental group (particles loaded 1, 2, 4, or 7 days prior). After 24 hours of culture, MSCs were lysed and RNA collected for quantitative real‐time polymerase chain reaction (qRT‐PCR) to measure IDO gene expression. RNA was extracted using an E.Z.N.A. Total RNA kit (OMEGA Bio‐Tek, Norcross, GA, http://www.omegabiotek.com/), and cDNA was subsequently synthesized (300 ng RNA per sample) with an iScript cDNA synthesis kit (Bio‐Rad, Hercules, CA, http://www.bio-rad.com/). Forward and reverse primers for *IDO1* (forward: AGCTTCGAGAAAGAGTTGAGAAG; reverse: GTGATGCATCCCAGAACTAGAC) and *18S* (forward: CTTCCACAGGAGGCCTACAC; reverse: CTTCGGCCCACACCCTTAAT) were designed by using Primer‐Blast ( http://www.ncbi.nlm.nih.gov) and purchased from Thermo Fisher. *IDO1* gene expression was calculated with respect to untreated MSCs and normalized to *18S* expression using the ΔΔCT method.

### MSC Spheroid Formation

**Figure 1:**
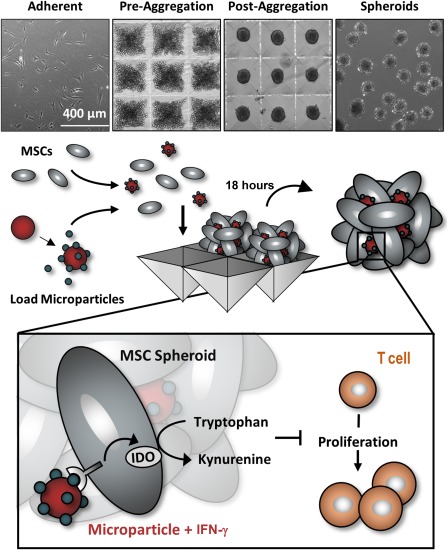
Microparticle delivery of interferon (IFN)‐γ within mesenchymal stem/stromal cell (MSC) spheroids. To form aggregates, we dissociated MSCs from adherent culture and mixed them with IFN‐γ‐loaded microparticles. Spheroids were subsequently formed via forced aggregation of cells and particles within microwell arrays to entrap cytokine‐loaded microparticles within the spheroidal construct to provide sustained local presentation of IFN‐γ within the MSC aggregates. Abbreviations: IFN, interferon; MSC, mesenchymal stem/stromal cell; IDO, indoleamine 2,3‐dioxygenase.

Three‐dimensional (3D) spheroids were formed by forced aggregation of MSCs into an array of 400 × 400 μm inverse pyramidal agarose microwells as a high throughput method of generating homogenous cell aggregates. For all experiments, 500‐cell spheroids were formed by adding 6 × 10^5^ cells to an agarose insert containing 1,200 microwells and centrifuging at 200× *g* for 5 minutes. After 18 hours, MSCs self‐assembled into spherical aggregates. In order to form spheroids with microparticles, we mixed a suspension of unloaded heparin microparticles or microparticles previously incubated with 33 or 333 ng IFN‐γ per 1 × 10^6^ MPs for 18 hours with the cell suspension at a 2:1 microparticle‐to‐MSC ratio and added to the microwells (Fig. 1). The incorporation efficiency of heparin microparticles within MSCs spheroids was quantified by lysing spheroids after initial formation and counting the number of particles retrieved from the spheroids. Furthermore, MSC spheroids without particles were also formed, and a subset was pretreated with IFN‐γ at equivalent doses to IFN‐γ microparticle groups (20 ng/ml or 200 ng/ml concentration, equivalent to 66 ng or 666 ng per 1 × 10^6^ cell, respectively). After 18 hours of microwell aggregate formation, spheroids were either cultured alone to assess IDO and immunomodulatory factor expression or with PBMCs to assess the immunomodulatory activity of MSC spheroids.

### Analysis of MSC Immunomodulatory Factor Expression

To evaluate MSC IDO expression in response to IFN‐γ pretreatment or microparticle delivery, we cultured MSC spheroids for up to 7 days. At days 1, 2, 4, and 7, MSCs were collected for qRT‐PCR analysis of *IDO1* gene expression in relation to untreated MSC spheroids. Cell lysates of MSC spheroids were also collected at days 1 and 4 for Western blotting. Sample protein concentration was first determined using a bicinchoninic acid assay Protein Quantification kit (Pierce Biotechnology, Rockford, IL, https://www.thermofisher.com). Sodium dodecyl sulfate polyacrylamide gel electrophoresis (SDS/PAGE) loading buffer was then mixed with 25 µg of protein per sample and incubated at 95°C for 5 minutes. Protein samples were subsequently loaded in 4%–15% Mini‐PROTEAN TGX gels (Bio‐Rad Laboratories). Vertical electrophoresis was performed using the Mini‐PROTEAN Tetra Cell (Bio‐Rad) system with SDS/PAGE running buffer at 200 V for 20 minutes with a 10‐ to 250‐kDa protein ladder (Precision Plus Protein Kaleidoscope; Bio‐Rad) as a molecular weight reference. Protein was then transferred to a nitrocellulose membrane (Bio‐Rad) via semidry transfer (Trans‐Blot SD; Bio‐Rad) at 25 V for 20 minutes. Membranes were blocked with IR blocking medium (Rockland Immunochemicals, Pottstown, PA, USA, https://www.rockland-inc.com) for 1 hour, followed by incubation with primary antibodies for IDO (rabbit anti‐IDO IgG; Abcam, Cambridge, U.K., http://www.abcam.com) and the loading control glyceraldehyde‐3‐phosphate dehydrogenase (GAPDH) (monoclonal goat anti‐GAPDH; Abcam) overnight at 4°C. Secondary IR antibodies (680 donkey anti‐rabbit and donkey 800 anti‐goat; Li‐Cor Biosciences, Lincoln, NE, https://www.licor.com) were used to detect IDO and GAPDH protein, respectively, and the blots were imaged using an Odyssey IR imager (LiCor Biosciences).

### Peripheral Blood Mononuclear Cell Cocultures

PBMCs were isolated from whole blood obtained with institutional review board approval from healthy volunteers via Ficoll (Sigma‐Aldrich) density gradient separation. Isolated PBMCs were cultured at 400,000 cells per well in 24‐well plates with MSC spheroids at ratios of 1:3, 1:1, and 3:1 MSCs‐to‐PBMCs, on the basis of previously reported ratios 18, 45, 46 and preliminary testing. At the start of coculture, 0.2 μg/ml of anti‐human CD3 and CD28 antibodies were added to the cocultures to induce T‐cell proliferation and activation. To evaluate microparticle delivery of IFN‐γ within MSC spheroids, we evaluated four experimental groups: (a) MSC spheroids without additional treatment, (b) spheroids pretreated with 200 ng/ml (666 ng per 1 × 10^6^ cells) of soluble IFN‐γ, (c) spheroids containing unloaded heparin microparticles, and (d) spheroids containing heparin microparticles (2:1 MP:MSC ratio) loaded with 333 ng IFN‐γ per 1 × 10^6^ particles. Where indicated, 1‐methyl_DL_‐tryptophan (1‐MT, diluted in 0.1 N NaOH; Sigma‐Aldrich), an inhibitor of IDO activity, or an equivalent volume of a vehicle control (0.1 N NaOH) was added to cocultures at a final concentration of 5 mM 1‐MT. In a subset of cultures, PBMCs were added to the upper well of a 0.4‐μm pore polycarbonate Transwell (Corning) and cocultured with MSC spheroids pretreated with 200 ng/ml IFN‐γ at a 3:1 MSC‐to‐PBMC ratio. MSC spheroids were added to PBMC cocultures immediately after aggregate formation, except where indicated otherwise. To test the persistence of spheroid immunomodulatory activity in culture, we added spheroids to PBMC cocultures 2 or 4 days after initial aggregate formation and IFN‐γ pretreatment. For all PBMC cocultures, T‐cell proliferation was assessed 4 days later by flow cytometry analysis of CD3 (fluorescein isothiocyanate‐conjugated mouse anti‐CD3 IgG; BD Biosciences, East Rutherford, NJ, https://www.bdbiosciences.com) and Ki67 (phycoerythrin‐conjugated mouse anti‐Ki67 IgG; BD Biosciences) double‐positive‐stained cells. T‐cell activation was also assessed after 4 days by measuring the amount of IFN‐γ in coculture spent media supernatants by ELISA (R&D). Finally, expression of anti‐inflammatory cytokines was assessed after 4 days by measuring the amount of IL‐10 in coculture spent media supernatants by ELISA (R&D).

Monocytes were isolated from PBMCs using an EasySep Human CD14^+^ Positive Selection Kit (STEMCELL Technologies) and cultured at 400,000 cells per well in 24‐well plates with MSC spheroids from each experimental group as in PBMC coculture experiments at a 3:1 MSC‐to‐monocyte ratio with no supplemented cytokines. After 4 days of coculture, monocyte phenotype was evaluated by determining the amounts of the proinflammatory cytokine, TNF‐α, and the anti‐inflammatory cytokine, IL‐10, in coculture spent media supernatants by ELISA (R&D).

### Statistical Analysis

Statistical analyses were performed using GraphPad Prism 6 software (Graphpad Software, La Jolla, CA, USA, http://www.graphpad.com/). Data are presented as mean ± SEM. Comparisons between multiple experimental groups were conducted using analysis of variance after Box‐Cox transformation to ensure normal distribution. Tukey post hoc analysis was then used to determine statistically significant differences between experimental groups, with a *p* value <.05, indicating significance.

## Results

### IFN‐γ Binds to Heparin Microparticles and Remains Bioactive During 1 Week of Culture

**Figure 2:**
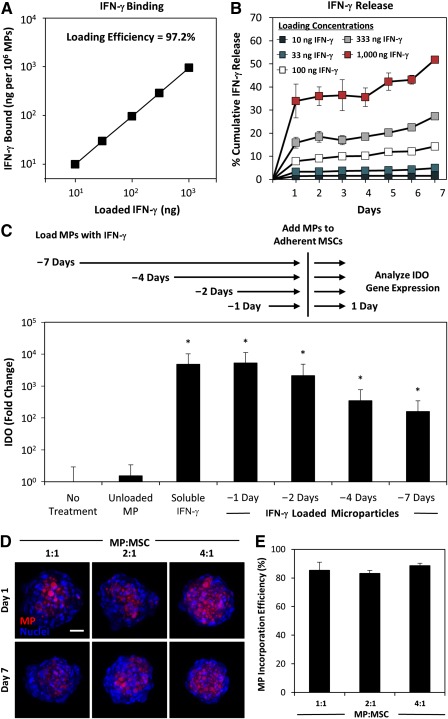
Heparin microparticles bind and present bioactive interferon (IFN)‐γ and incorporate stably within mesenchymal stem/stromal cell (MSC) aggregates. **(A):** Heparin microparticles bound >97% of IFN‐γ from solution over the ranges tested. **(B):** Although an initial burst release of IFN‐γ from the particles was observed, little IFN‐γ was subsequently released from the particles during 7 days. **(C):** Particle‐bound IFN‐γ remained bioactive and capable of inducing MSC expression of indoleamine 2,3‐dioxygenase during 7 days. **(D):** Fluorescently labeled microparticles were incorporated within MSC aggregates at various doses and remained stably incorporated for at least 7 days of culture. Maximal intensity projection, scale bar = 50 μm. **(E):** Approximately 85% of microparticles added during aggregate formation were incorporated within MSC spheroids within the range of microparticle‐to‐cell ratios tested, allowing for precise control of microparticle and loaded cytokine dose. ∗*p* < .05, in comparison with no treatment. Abbreviations: IDO, indoleamine 2,3‐dioxygenase; IFN, interferon; MP, microparticle; MSC, mesenchymal stem/stromal cell.

To establish microparticle‐loading conditions for delivery of IFN‐γ within MSC spheroids, we first investigated the interaction between heparin microparticles and IFN‐γ. Heparin particles have previously been characterized as having a large binding capacity for the growth factor bone morphogenetic protein (BMP‐2) (∼300 µg per milligram of MPs 38). When heparin particles were incubated in solutions of varying concentrations of IFN‐γ (10–1,000 ng per 1 × 10^6^ particles; equivalent to 0.2–20 µg per milligram of MPs), more than 97% of the initial IFN‐γ was depleted from solution after 18 hours for each concentration tested (Fig. 2A). However, the maximum binding capacity of the microparticles for IFN‐γ was not reached within the range of concentrations tested, and the highest concentration of IFN‐γ tested (approximately 20 µg IFN‐γ per milligram of MPs) was significantly lower than the maximal binding capacity of heparin microparticles for BMP‐2 (300 µg BMP‐2 per milligram of MPs). Therefore, if desired, even greater amounts of IFN‐γ could likely be loaded onto the particles.

After loading particles with different concentrations of IFN‐γ, we resuspended microparticles in fresh cell culture medium containing 10% FBS and incubated for 1 week to examine release of IFN‐γ from the particles. At each loading concentration, the majority of IFN‐γ released from the particles occurred within the first 24 hours, suggesting an initial burst release of loosely bound IFN‐γ from the particles (Fig. 2B). The amount of IFN‐γ released during this 24‐hour period was dependent on the loading concentration, with approximately 1% of loaded IFN‐γ at the lowest loading concentration and approximately 30% of loaded IFN‐γ at the highest loading concentration. However, after the initial burst release period, minimal IFN‐γ was released from the particles during the next 6 days, suggesting that the majority of loaded IFN‐γ remained bound to the heparin microparticles for at least 1 week of culture.

The bioactivity of IFN‐γ bound to heparin microparticles was evaluated by culturing adherent MSCs in direct contact with microparticles loaded with IFN‐γ (333 ng per 1 × 10^6^ MPs). Microparticles were loaded with IFN‐γ 1, 2, 4, and 7 days prior to addition to MSC cultures to examine whether the bioactivity of microparticle‐bound IFN‐γ was retained over time. The ability of particle‐bound IFN‐γ to induce IDO expression in MSCs was compared with treatment with unloaded microparticles or an equivalent dose of soluble IFN‐γ. As was expected, treatment of adherent MSCs with IFN‐γ induced IDO expression (>10^3^‐fold increase in IDO gene expression; *p* < .001), whereas the addition of unloaded microparticles did not significantly increase MSC IDO expression (Fig. 2C). When an equivalent dose of IFN‐γ was delivered via microparticles loaded 1 day prior to culture, IDO expression was induced in adherent MSCs to the same extent as was soluble treatment, demonstrating that microparticle‐bound IFN‐γ remains bioactive. Furthermore, microparticles loaded with IFN‐γ 2, 4, and 7 days prior to addition to MSCs maintained the ability to significantly induce MSC IDO expression (2000‐, 350‐, and 150‐fold increase in IDO expression, in comparison with untreated MSCs, respectively; *p* < .001), although microparticles loaded with IFN‐γ 4 and 7 days prior to addition to MSCs induced IDO at a somewhat lower magnitude in comparison with freshly loaded microparticles (15‐ and 30‐fold decrease in IDO expression, respectively; *p* < .001). Because minimal IFN‐γ is released from heparin microparticles following the first 24 hours, these results suggest that IFN‐γ loaded onto microparticles retains its bioactivity and ability to elicit a functional MSC response while bound to heparin for at least 1 week of culture.

### IFN‐γ‐Loaded Microparticles Induce Sustained Immunomodulatory Factor Expression

To evaluate incorporation of heparin microparticles within aggregates, we incorporated fluorescently labeled, unloaded microparticles within MSC aggregates at several different ratios of microparticles to MSCs (1:1, 2:1, and 4:1 MP:MSC) and cultured for up to 7 days to evaluate the incorporation efficiency of particles within aggregates and retention of particles within the multicellular constructs. Fluorescently labeled microparticles were readily incorporated within MSC spheroids after initial formation and remained stably incorporated within spheroids for at least 1 week of culture (Fig. 2D). Approximately 85% of the particles initially added during aggregate formation were incorporated within spheroids for all three ratios tested (Fig. 2E). Additionally, only ∼1.5% of these particles were found to escape from the spheroids into the culture medium during 1 week of culture. By determining the incorporation efficiency of microparticles within MSC spheroids (∼85%) and persistence of particles within spheroids during the course of 7 days, we were able to adjust the number of particles added at the time of formation for subsequent experiments to ensure that equivalent doses of IFN‐γ presented via particles in comparison with soluble pretreatment with IFN‐γ.

**Figure 3:**
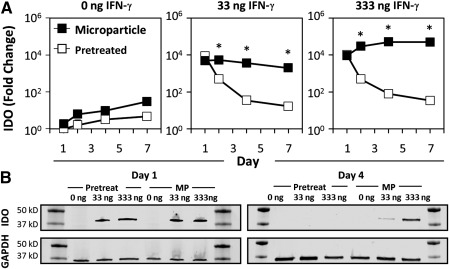
Interferon (IFN)‐γ‐loaded heparin microparticles induce sustained IDO expression. **(A):** IDO gene expression rapidly decreased after initial pretreatment of mesenchymal stem/stromal cell (MSC) spheroids with IFN‐γ, whereas IDO gene expression was sustained during 7 days when IFN‐γ was delivered by microparticles within MSC spheroids. ∗*p* < .05, comparing pretreatment and microparticle treatment on the same day of culture. **(B):** IDO protein expression was induced by IFN‐γ but was only expressed by MSC spheroids after 4 days when IFN‐γ was delivered by microparticles. Abbreviations: GAPDH, glyceraldehyde‐3‐phosphate dehydrogenase; IDO, indoleamine 2,3‐dioxygenase; IFN, interferon; MP, microparticle.

To investigate whether sustained delivery of IFN‐γ directly within the MSC spheroidal microenvironment could enhance MSC immunomodulatory factor expression, we loaded heparin particles with two doses of IFN‐γ (33 ng and 333 ng IFN‐γ per 1 × 10^6^ MPs) or without any cytokine and incorporated into MSC spheroids. The expression of IDO by MSC spheroids with microparticles or pretreated with IFN‐γ was then measured during 1 week of culture. Little change in IDO gene expression was observed in MSC spheroids without IFN‐γ pretreatment or with unloaded microparticles during 1 week (Fig. 3A). Both IFN‐γ pretreatment and incorporation of IFN‐γ‐loaded microparticles initially induced IDO gene expression within the first day (approximately 10^4^‐fold; *p* < .001). However, IDO expression rapidly decreased when MSC spheroids were pretreated with soluble IFN‐γ. In contrast, MSC spheroids with IFN‐γ− loaded microparticles maintained an elevated level of IDO gene expression during an entire week of culture. Similarly, protein expression of IDO was detected initially on day 1 during which MSC spheroids were exposed to IFN‐γ by pretreatment or via microparticles (Fig. 3B). By day 4, only spheroids exposed to the sustained presentation of microparticle‐bound IFN‐γ continued to express elevated levels of IDO. Finally, no IFN‐γ was detected in the spent cell culture medium by ELISA, indicating that little to no microparticle‐bound IFN‐γ escaped from the spheroids (<0.1% of IFN‐γ initially loaded onto particles on the basis of the lower detection limit of ELISA).

### IFN‐γ‐Loaded Microparticles Enhance MSC Suppression of T‐Cell Activation and Proliferation

**Figure 4:**
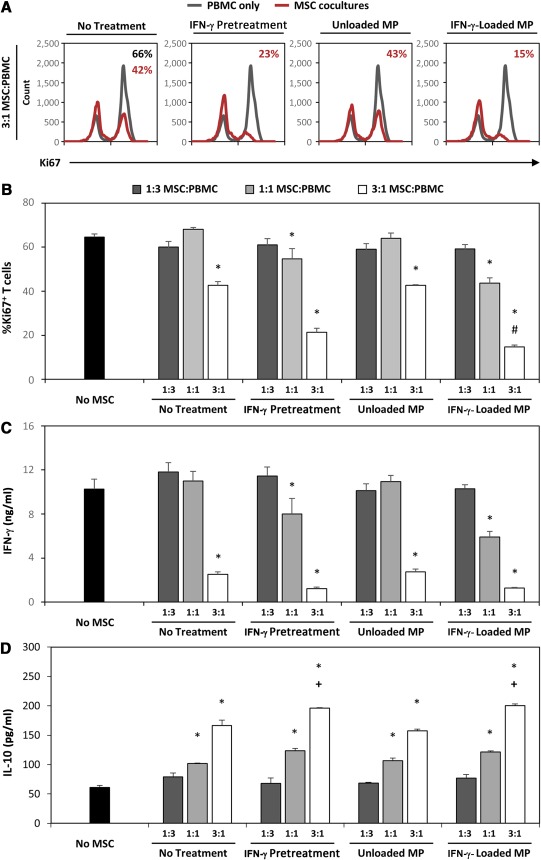
Microparticle delivery of interferon (IFN)‐γ enhances mesenchymal stem/stromal cell (MSC) spheroid suppression of T‐cell activation and proliferation. **(A):** Representative histograms of Ki67^+^ T‐cells in coculture with MSC spheroids at 3:1 MSC:peripheral blood mononuclear cell (PBMC) ratios demonstrate increased suppression of T‐cell proliferation when treated with IFN‐γ via pretreatment or microparticle delivery. **(B):** Coculture of activated PBMCs with IFN‐γ‐loaded microparticle‐treated MSC spheroids resulted in the greatest suppression of T‐cell proliferation at a 3:1 MSC:PBMC ratio. Only MSCs treated with IFN‐γ (via pretreatment or microparticle) suppressed T‐cell proliferation at a 1:1 MSC:PBMC ratio. **(C):** MSC spheroids suppressed expression of the effector cytokine IFN‐γ, and the greatest suppression was observed in cocultures with MSCs treated with IFN‐γ. **(D):** IL‐10 expression was increased in cocultures with MSC spheroids and was greatest when MSCs were treated with IFN‐γ via pretreatment or microparticle delivery. ∗, *p* < .05 in comparison with No‐MSC cultures; #, *p* < .05 in comparison with IFN‐γ‐pretreated MSC spheroids at the same MSC:PBMC ratio; +, *p* < .05 in comparison with No Treatment and Unloaded Microparticle MSC spheroid cocultures. Abbreviations: IFN, interferon; IL, interleukin; MP, microparticle; MSC, mesenchymal stem/stromal cell; PBMC, peripheral blood mononuclear cell.

Because microparticle delivery of IFN‐γ promoted sustained MSC expression of IDO, we next sought to determine whether the enhanced IDO expression resulted in increased suppression of T‐cell proliferation and activation. At the highest ratio of MSCs to PBMCs (3: 1 MSC:PBMC), suppression of T‐cell proliferation, as measured by decreased Ki67 expression, was observed in all MSC coculture groups in comparison with the activated PBMCs alone (Fig. 4A). However, MSC spheroids pretreated with IFN‐γ or incorporated with IFN‐γ‐loaded microparticles induced greater suppression of T‐cell proliferation than did untreated MSC spheroids. Furthermore, only MSCs treated with IFN‐γ (either through pretreatment or microparticles) were capable of suppressing T‐cell proliferation at a 1:1 MSC:PBMC ratio. Additionally, the sustained microparticle delivery of IFN‐γ within spheroids significantly enhanced suppression of T‐cell proliferation at both a 3:1 ratio and 1:1 ratio of MSCs:PBMCs in comparison with spheroids pretreated with IFN‐γ (Fig. 4B). At a 3:1 MSC:PBMC ratio, only 15% ± 1% of T cells were Ki67+ in coculture with MSCs with IFN‐γ‐loaded microparticles in comparison with 23% ± 2% in cocultures with pretreated MSC spheroids (*p* = .029). No suppression of T‐cell proliferation was observed when MSCs were cocultured at a 1:3 MSC:PBMC ratio in any of the IFN‐γ treatment groups. Finally, a similar response was observed in expression of IFN‐γ and IL‐10 in cocultures. IFN‐γ and IL‐10 detected in MSC‐PBMC cocultures were expressed by cells from the PBMC fraction, because no IFN‐γ or IL‐10 could be detected in cultures of only MSC spheroids with or without IFN‐γ‐loaded microparticles. The greatest suppression of IFN‐γ secretion was observed in cocultures with pretreated MSC spheroids and MSC spheroids with IFN‐γ‐loaded microparticles (Fig. 4C). Similarly, IL‐10 expression was increased in cocultures with MSC spheroids at 3:1 and 1:1 MSC:PBMC ratios and was greatest in MSC spheroids pretreated with IFN‐γ or incorporated with IFN‐γ‐loaded microparticles (Fig. 4D).

### Microparticle‐Induced Suppression of T‐Cell Activation and Proliferation Is Dependent on Induction of MSC IDO Expression and Aided by Anti‐Inflammatory Monocytes

**Figure 5:**
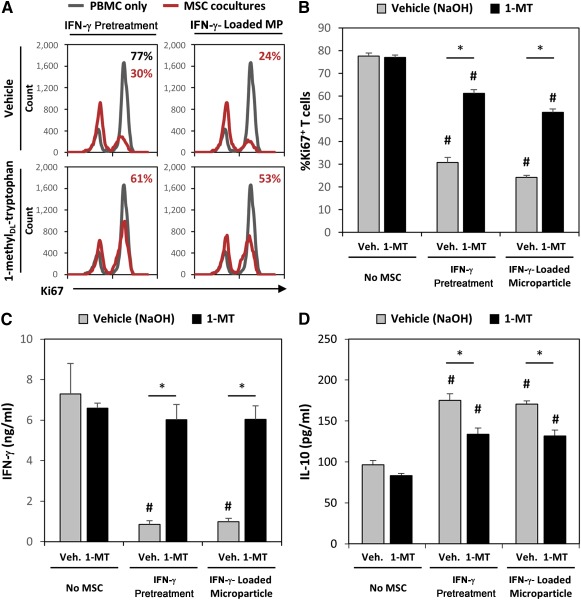
Increased suppression of T‐cells by microparticle delivery of interferon (IFN)‐γ is dependent on induction of indoleamine 2,3‐dioxygenase (IDO) expression. **(A):** Representative histograms of Ki67^+^ T‐cells in coculture with IFN‐γ‐pretreated or IFN‐γ‐loaded microparticle‐ treated MSC spheroids with or without the IDO inhibitor 1‐methyl_DL_‐tryptophan (1‐MT). **(B):** Addition of 1‐MT to mesenchymal stem/stromal cell (MSC)‐peripheral blood mononuclear cell (PBMC) coculture (3:1 MSC:PBMC ratio) significantly inhibited the ability of IFN‐γ‐primed MSCs to inhibit T‐cell proliferation in both pretreated and microparticle‐treated spheroids. Inhibition of IDO activity with 1‐MT increased T‐cell expression of IFN‐γ **(C)** and reduced the induction of interleukin‐10 expression in PBMC cocultures **(D)**. ∗, *p* < .05 in comparison with groups denoted by bar. #, *p* < .05 in comparison with No‐MSC cultures. Abbreviations: IFN, interferon; IL‐10, interleukin‐10; MSC, mesenchymal stem/stromal cell; PBMC, peripheral blood mononuclear cell; Veh., vehicle; 1‐MT, 1‐methyl_DL_‐tryptophan.

To determine whether the IDO expression in response to IFN‐γ‐loaded microparticles was responsible for the enhanced ability of MSCs to suppress T cells, we cultured pretreated and microparticle‐treated MSC spheroids with activated PBMCs in the presence of an IDO inhibitor, 1‐methyl_DL_‐tryptophan (1‐MT). Supplementation of 1‐MT into cocultures significantly impaired the ability of IFN‐γ‐pretreated spheroids and spheroids with IFN‐γ‐loaded microparticles to suppress T‐cell proliferation (Fig. 5A). In PBMC cocultures with IFN‐γ‐pretreated spheroids, 61% ± 2% of T cells were Ki67^+^ when 1‐MT was added to the culture in comparison with 31% ± 2% Ki67^+^ T cells in cocultures with the vehicle control (*p* < .001). Similarly, 57% ± 2% of T cells were Ki67^+^ in cocultures with MSC spheroids with IFN‐γ‐loaded microparticles and 1‐MT in comparison with 24% ± 1% Ki67^+^ T cells in cocultures treated with the vehicle control (*p* < .001). However, even with supplementation of 5 µM 1‐MT into cocultures, both pretreated and microparticle‐treated spheroids were capable of slightly suppressing T‐cell proliferation in comparison with activated PBMCs alone (77% ± 2% Ki67^+^, *p* < .001). MSC suppression of T‐cell IFN‐γ expression was completely inhibited by treatment with 1‐MT in cocultures with IFN‐γ−pretreated MSC spheroids and spheroids with IFN‐γ‐loaded microparticles (Fig. 5C). However, PBMC expression of IL‐10 in response to MSC spheroids was only partially inhibited by 1‐MT (Fig. 5D; 133 and 132 pg/ml of IL‐10 in pretreated and microparticle groups in comparison with 83 pg/ml of IL‐10 in No‐MSC− 1‐MT control; *p* = .003 and .004, respectively). In addition to IDO, direct cell‐contact mechanisms, mediated by PDL1/2 or FasL signaling, can play a role in aiding suppression of T cells 15, 16. Physical separation of IFN‐γ‐stimulated MSC spheroids from PBMCs significantly reduced the ability of MSCs to suppress T‐cell proliferation ( supplemental online Fig. 1; ∼35% Ki67^+^ T cells in direct coculture, ∼52% Ki67^+^ in Transwell coculture; *p* = .022). Although additional immunomodulatory factors may contribute to the residual suppressive effects observed of MSC spheroids treated with 1‐MT, IDO inhibition significantly impairs the ability of MSCs to suppress T‐cell proliferation (Fig. 5B), as was expected on the basis of previous studies 17, 18.

**Figure 6:**
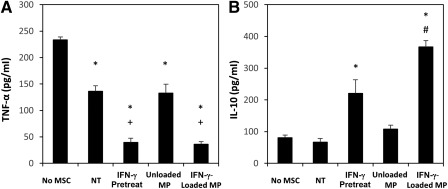
Microparticle delivery of interferon (IFN)‐γ increases mesenchymal stem/stromal cell (MSC) spheroid polarization of monocytes to anti‐inflammatory phenotypes. **(A):** Monocyte expression of tumor necrosis factor‐α, a proinflammatory cytokine, was decreased in all cocultures with MSC spheroids but was greatest in cultures with IFN‐γ pretreated or microparticle‐treated MSCs. **(B):** Conversely, interleukin‐10 production, an anti‐inflammatory cytokine, was greatest in cocultures with IFN‐γ‐loaded microparticle‐treated MSCs and not increased in cocultures with untreated MSCs. Polarization of monocytes to anti‐inflammatory phenotypes by MSCs treated with IFN‐γ‐loaded microparticles can aid in the suppression of T‐cell proliferation and activation in peripheral blood mononuclear cell cocultures. ∗, *p* < .05 in comparison with No‐MSC group; #, *p* < .05 in comparison with IFN‐γ‐pretreated MSC spheroid group; +, *p* < .05 in comparison with No‐Treatment group. Abbreviations: IFN, interferon; IL‐10, interleukin‐10; MSC, mesenchymal stem/stromal cell; MP, microparticle; NT, no treatment; TNF‐α, tumor necrosis factor‐α.

In addition to IDO activity, MSC suppression of T‐cell proliferation and activation in PBMC coculture assays in vitro is also dependent on MSC induction of IL‐10‐expressing monocytes within these cocultures 18, 45, 46. Therefore, we investigated whether MSC spheroids containing IFN‐γ‐loaded microparticles could also suppress monocyte secretion of proinflammatory TNF‐α and increase monocyte secretion of anti‐inflammatory IL‐10 expression in direct cocultures with monocytes isolated from PBMCs. Monocyte secretion of TNF‐α was suppressed in coculture with MSC spheroids (Fig. 6A; ∼135 ng/ml TNF‐α in MSC spheroid coculture in comparison with ∼230 ng/ml in monocyte‐only culture). However, pretreatment of MSCs with IFN‐γ or delivery of IFN‐γ‐laden microparticles within MSC spheroids increased MSC suppression of monocyte TNF‐α expression (∼40 ng/ml TNF‐α in coculture with IFN‐γ− pretreated or IFN‐γ‐loaded microparticle‐treated MSCs). Additionally, the amount of the immunoregulatory cytokine IL‐10 in spent media supernatants was greatest in cocultures with spheroids containing IFN‐γ‐loaded microparticles (Fig. 6B), suggesting that the increase in monocyte IL‐10 expression and decrease in TNF‐α may be aiding the suppression of T cells observed in cocultures with spheroids containing IFN‐γ‐loaded microparticles.

### Microparticle Delivery of IFN‐γ Within MSC Spheroids Sustains Immunomodulatory Activity in Comparison With Soluble Pretreated MSCs

**Figure 7:**
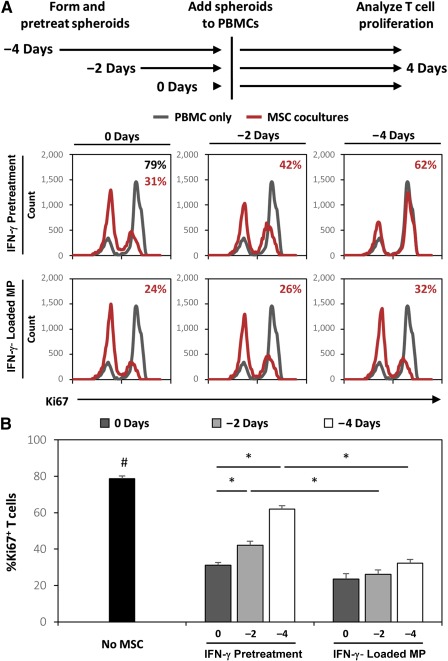
Microparticle delivery of interferon (IFN)‐γ enhances mesenchymal stem/stromal cell (MSC) suppression of T‐cells for more than 1 week. **(A):** Representative histograms of Ki67+ T‐cells in coculture with MSC spheroids treated with IFN‐γ or IFN‐γ‐loaded microparticles 0, 2, and 4 days prior to addition to peripheral blood mononuclear cell coculture. **(B):** IFN‐γ‐loaded microparticles sustained the enhanced MSC suppression of T‐cell proliferation in response to IFN‐γ for up to 8 days, whereas the effects of pretreating MSC spheroids with IFN‐γ were transient and resulted in decreased ability to suppress T‐cell proliferation over time. ∗, *p* < .05 in comparison with groups denoted by bars; #, *p* < .05 in comparison with No‐MSC group. Abbreviations: IFN, interferon; MP, microparticle; MSC, mesenchymal stem/stromal cell; PBMC, peripheral blood mononuclear cell.

Because MSC spheroids with IFN‐γ‐loaded microparticles expressed elevated levels of IDO for at least 7 days and IDO activity of pretreated MSC spheroids decreased rapidly for 4 days, we investigated whether MSC spheroids containing IFN‐γ‐loaded microparticles continued to modulate T‐cell responses even 4 days after formation. Therefore, MSC spheroids were pretreated or formed with IFN‐γ‐loaded microparticles and added to cocultures with activated PBMCs after 0, 2, or 4 days following initial aggregate formation and pretreatment. Consistently, both spheroids pretreated with IFN‐γ and spheroids with IFN‐γ‐loaded microparticles suppressed T‐cell proliferation when added to cocultures immediately after spheroid formation (Fig. 7A). However, when spheroids were added 2 days after formation and IFN‐γ pretreatment, the ability of pretreated spheroids to suppress T‐cell proliferation was significantly reduced (*p* = .035 pretreated spheroid days 0–4 vs. pretreated spheroid days 2–6). Conversely, no difference in T‐ cell suppression ability was observed between spheroids added immediately after formation or 2 days after formation, in those spheroids containing IFN‐γ‐loaded microparticles (*p* = .978 spheroids with IFN‐γ‐loaded particles days 0–4 vs. spheroids with IFN‐γ‐loaded particles days 2–4). Moreover, the ability of spheroids pretreated with IFN‐γ to suppress T‐cell proliferation was further decreased when spheroids were added 4 days after initial formation and pretreatment. The extent of T‐cell suppression observed in pretreated spheroids after 4 days was comparable to levels observed previously when nontreated MSC spheroids were cocultured with activated PBMCs, suggesting that the effects of pretreatment were completely abolished within 4 days. However, MSCs with IFN‐γ‐loaded microparticles retained the ability to suppress T‐cell proliferation even when added to PBMC cocultures 4 days after initial formation (Fig. 7B). Altogether, MSC spheroids incorporated with IFN‐γ microparticles exhibit a sustained ability to suppress T‐cell proliferation during at least 1 week of culture, whereas the immunosuppressive ability of MSC spheroids pretreated with IFN‐γ rapidly decreased.

## Discussion

In this study, we designed and evaluated a strategy for locally presenting IFN‐γ within MSC constructs to potentiate sustained MSC immunomodulatory activity. To do so, we took advantage of a microparticle‐based approach previously developed to spatially and temporally control presentation of growth factors within stem cell aggregates 34 35 36 37. Heparin‐based microparticles were chosen to deliver IFN‐γ to exploit the high native affinity of IFN‐γ for heparin and the ability of heparin to maintain protein bioactivity. These heparin particles have previously been demonstrated to stably bind positively charged heparin‐binding growth factors including BMP‐2, vascular endothelial growth factor, and fibroblast growth factor 2 38. Consistent with previous studies, heparin microparticles bound large amounts of IFN‐γ (>20 µg per mg MPs), and very little IFN‐γ was released from the particles after the initial burst release period during the first 24 hours postloading. Despite low IFN‐γ release, IFN‐γ− loaded particles were still capable of inducing MSC IDO expression even after 7 days of incubation at physiologic conditions prior to addition to MSCs. These results are consistent with previous studies investigating the interaction between cell‐derived heparan sulfate and IFN‐γ signaling. Heparan sulfate present within plasma membranes can augment IFN‐γ signaling by stably immobilizing IFN‐γ at the cell surface in close proximity to cell membrane receptors 42. In contrast, supplementation of soluble heparin into culture medium can suppress the activity of IFN‐γ, likely because of sequestration of IFN‐γ in solution that inhibits its interaction with cell surface receptors. Altogether, these results suggest that the ability of IFN‐γ‐loaded microparticles to induce MSC IDO expression is dependent on direct contact between particle‐bound IFN‐γ and MSCs and does not require the release of soluble IFN‐γ from the particles. Furthermore, these results also corroborate results found in previous studies demonstrating that physical separation of cells and growth factor‐loaded heparin microparticles via Transwell significantly reduces the activity of particle‐bound growth factors 38. Additionally, because heparin‐bound IFN‐γ bioactivity is mediated primarily through solid‐phase presentation of IFN‐γ and not soluble release, there is little risk of exogenous IFN‐γ escaping MSC spheroids and exacerbating an inflammatory response in vivo. Overall, these results demonstrate that heparin microparticles are an effective carrier for locally restricting sustained delivery of bioactive IFN‐γ within MSC spheroids.

Pretreatment of MSCs with inflammatory cytokines is frequently used to enhance the immunomodulatory activity of the cells by activating expression of IDO and other immunomodulatory factors prior to injection. However, we have demonstrated herein that sustained presentation of IFN‐γ to MSCs enhances and extends the duration of immunomodulatory activity by MSCs. Using a heparin microparticle‐based strategy, expression of IDO by MSCs was sustained for at least 1 week. Furthermore, microparticle delivery of IFN‐γ within MSC spheroids increased MSC suppression of T‐cell proliferation and activation in comparison with pretreated spheroids. By locally concentrating IFN‐γ within the MSC spheroid through particle‐mediated delivery, MSCs may be exposed to an effectively higher dose of cytokine in relation to an equal soluble amount, thereby resulting in enhanced immunomodulatory activity. More strikingly, incorporation of IFN‐γ‐loaded microparticles within MSC spheroids significantly extends the duration of MSC immunomodulation. Although the immunomodulatory activity of pretreated MSC spheroids was significantly reduced after 2 days, MSC spheroids with IFN‐γ‐loaded microparticles continued to effectively suppress T cells through 1 week of culture. Not surprisingly, on the basis of the targeted strategy we used, immunosuppressive effects of MSCs were mediated by IDO expression because the IDO inhibitor 1‐MT reduced the ability of MSCs to suppress T‐cell proliferation. Although IDO expression is necessary for human MSC immunomodulation, additional MSC secreted factors or direct cell contact‐mediated mechanisms may also play an important role in the suppression of T‐cell proliferation. In addition to direct modulation of T cells by MSCs, MSC induction of IL‐10‐ expressing monocytes within PBMC cocultures also aids in the suppression of T cells in vitro 18. Therefore, the increased induction of IL‐10‐expressing monocytes by MSC spheroids loaded with IFN‐γ microparticles could also facilitate the increased immunosuppressive activity of 3D MSC constructs.

Despite the extensive use of biomaterials with specific biophysical and biochemical properties and ability to deliver biochemical stimuli to direct differentiation of MSCs, similar approaches have largely been unexplored for directing the immunomodulatory paracrine activity and broader trophic effects of MSCs. Numerous studies have demonstrated that MSC paracrine factor secretion is modulated in response to environmental cues in addition to the local cytokine milieu, including matrix composition, mechanics, and topography 47, 48, as well as oxygen tension 49. Furthermore, environmental stimuli are highly amenable to manipulation via biomaterials to specifically define the microenvironment of stem cells and direct their function 50. To date, only one biomaterial‐based approach has been used to increase MSC expression of IDO to enhance suppression of T‐cell proliferation and activation. Delivery of glucocorticoids within MSCs via poly‐lactic‐coglycolic acid nanoparticles internalized by MSCs enhanced the upregulation of IDO in response to IFN‐γ and resulted in increased suppression of CD3/CD28‐activated PBMCs 51. However, IDO expression by MSCs was still dependent on the presence of soluble IFN‐γ, and the glucocorticoid budesonide was unable to stimulate IDO alone. Therefore, sustained presentation of IFN‐γ and glucocorticoids may be complementary approaches for enhancing MSC immunomodulatory activity. Ultimately, engineering the local environment of transplanted MSCs through biomaterial‐based approaches provides an additional level of control over the efficacy of MSC therapies to promote maximal activity posttransplantation.

Overall, the approach described in this study provides a novel means of enhancing MSC immunomodulatory activity by locally concentrating and sustaining presentation of immunomodulatory stimuli and represents a viable method of potentiating MSC immunomodulation after local injection in vivo. Because MSCs are highly dependent on local inflammatory signals to stimulate their immunomodulatory activity, spatially controlled presentation of inflammatory cues within MSC aggregates provides a means of stimulating these cells even when environmental inflammatory signals may not be present, such as in systemic delivery strategies, states of chronic inflammation, or when used in combination with traditional anti‐inflammatory drugs. For example, although MSCs have been effective in treating graft‐versus‐host disease (GvHD) when infused at the height of inflammation, they are much less effective if delivered prior to the onset of inflammation 52, 53. Similarly, MSCs are less effective at treating experimental autoimmune encephalomyelitis when administered during disease remission 14, 54. In clinical studies, MSC‐based therapies have been most effective in treating immunosuppressant‐refractory acute inflammatory diseases 55, 56. At low concentrations of strong inflammatory signals (i.e., IFN‐γ or TNF‐α) present during chronic inflammatory states, MSCs upregulate chemokines that recruit lymphocytes 24, 57. However, low cytokine concentrations are not sufficient to induce substantial expression of IDO by MSCs to suppress lymphocyte activation and therefore can actually augment inflammation. Such studies highlight the critical importance of threshold concentrations of inflammatory stimuli being needed to effectively stimulate MSC immunomodulation. By presenting a sustained, high concentration of a cytokine, such as IFN‐γ, through incorporation of microparticles within MSC aggregates, the use of MSCs for in vivo immunomodulatory applications could be greatly expanded; for example, MSCs could potentially be transplanted concurrently with graft tissue to effectively suppress an immune response prior to the development of GvHD and severe inflammation. Furthermore, based on evidence that MSCs administered together with steroids or cyclosporine to immunosuppressant‐responsive patients have no clinical effect 58, 59, it is speculated that the lack of immunosuppression may be due to the decreased availability of inflammatory cytokines needed to stimulate MSC immunomodulation 60. Hence, in such a situation, locally sustained presentation of IFN‐γ to MSCs may improve outcomes in patients receiving both MSCs and traditional immunosuppressants by stimulating IDO expression despite the lack of systemic cytokines present in the host environment. Altogether, sustained presentation of IFN‐γ within MSC aggregates may provide a clinically relevant approach for inducing and enhancing MSC immunomodulatory activity in disease states previously deemed to be unresponsive to MSC‐based therapies.

## Conclusion

The results of this study demonstrate that heparin microparticle delivery of IFN‐γ within spheroidal MSC aggregates presents bioactive IFN‐γ locally within the MSC microenvironment that in turn induces a sustained MSC suppression of CD3/CD28‐activated T cells in vitro. The enhanced T‐cell suppression by MSC spheroids containing IFN‐γ‐loaded microparticles is mediated, in part, through upregulation of IDO expression and increasing monocyte IL‐10 expression. Overall, this approach could aid in overcoming the limitations of transient pretreatment strategies by continuously presenting bioactive IFN‐γ within the MSC microenvironment and thereby maximizing the immunomodulatory potential of transplanted MSCs. Furthermore, this approach provides a means of inducing potent MSC immunomodulation, irrespective of unknown or ill‐defined environmental inflammatory milieu, such as in chronic inflammation or when administered with immunosuppressants. Altogether, biomaterial‐based engineering of the MSC microenvironment provides a means of controlling the function of transplanted cells to specifically direct their therapeutic activity and may improve MSC‐based therapies aimed at treating a number of inflammatory and immune diseases.

## Author Contributions

J.A.Z.: conception and design, collection and/or assembly of data, data analysis and interpretation, manuscript writing; M.H.H.: conception and design, data analysis and interpretation, manuscript writing; T.C.M.: conception and design, financial support, data analysis and interpretation, manuscript writing, final approval of manuscript.

## Disclosure of Potential Conflicts of Interest

The authors indicated no potential conflicts of interest.

## Supporting information

Supporting Information
