# Supplementary material for: Enhanced Immunosuppression of T Cells by Sustained Presentation of Bioactive Interferon‐γ Within Three‐Dimensional Mesenchymal Stem Cell Constructs
Source: Stem Cells Transl Med. 2016 Aug 8;6(1):223–37. doi: 10.5966/sctm.2016-0044 (PMC5442746; doi:10.5966/sctm.2016-0044)
Supplement: Supplementary file 1 — Supporting Information [file SCT3-6-223-s001.pdf]

Supplemental Figure – McDevitt et al.

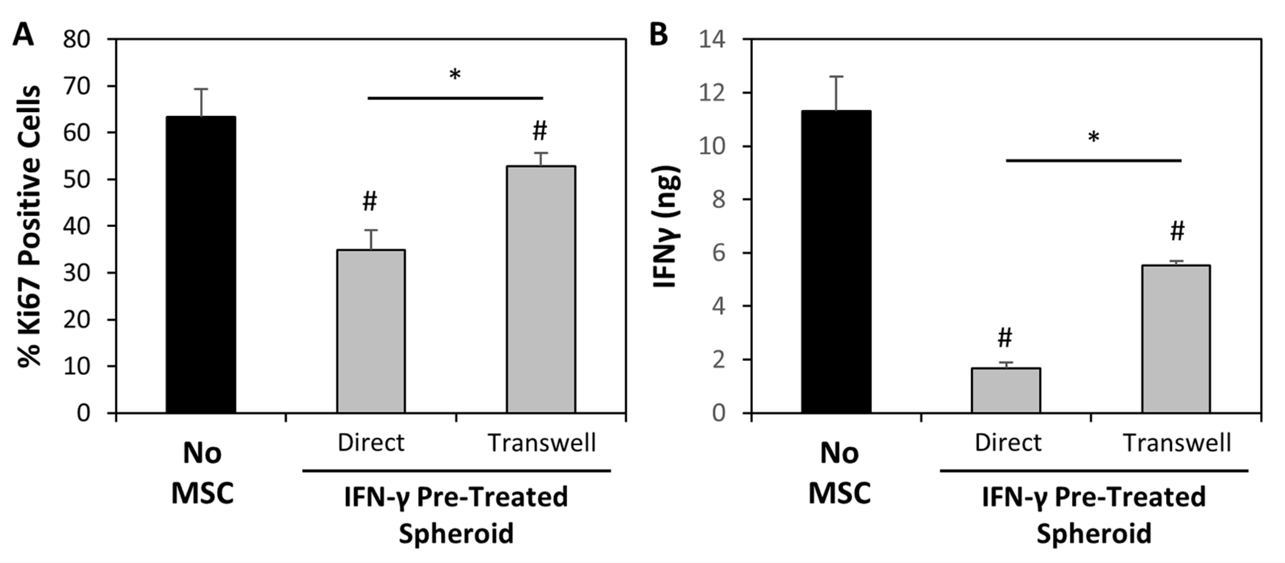

Figure S1. Transwell co-culture reduces the ability of spheroid MSCs to suppress T-cell proliferation. (A) Co-culture of CD3/CD28 activated PBMCs with spheroid MSCs in transwell co-culture reduced the ability of MSCs to suppress T-cell proliferation as determined by percentage of $Ki67^+$ T-cells. (B) Similarly, transwell co-culture reduced the ability of MSCs to suppress T-cell expression of the effector cytokine IFN-γ. * indicates $p < 0.05$ compared to groups denoted by bars. # indicates $p < 0.05$ compared to "No MSC" cultures.
